# Validation of thermal dynamics during Hyperthermic IntraPEritoneal Chemotherapy simulations using a 3D-printed phantom

**DOI:** 10.3389/fonc.2023.1102242

**Published:** 2023-02-14

**Authors:** Daan R. Löke, H. Petra Kok, Roxan F. C. P. A. Helderman, Nicolaas A. P. Franken, Arlene L. Oei, Jurriaan B. Tuynman, Remko Zweije, Jan Sijbrands, Pieter J. Tanis, Johannes Crezee

**Affiliations:** ^1^ Department of Radiation Oncology, Cancer Center Amsterdam, Amsterdam University Medical Centers (UMC), University of Amsterdam, Amsterdam, Netherlands; ^2^ Laboratory for Experimental Oncology and Radiobiology, Center for Experimental and Molecular Medicine, Cancer Center Amsterdam, University of Amsterdam, Amsterdam, Netherlands; ^3^ Department of Surgery, Amsterdam University Medical Centers (UMC), Vrije Universiteit Amsterdam, Cancer Center Amsterdam, Amsterdam, Netherlands; ^4^ Department of Surgery, Amsterdam University Medical Centers (UMC), University of Amsterdam, Cancer Center Amsterdam, Amsterdam, Netherlands; ^5^ Department of Surgical Oncology and Gastrointestinal Surgery, Erasmus Medical Center (MC), Rotterdam, Netherlands

**Keywords:** hyperthermic intrapertioneal chemotherapy (HIPEC), computational fluid dynamics (CFD), computational modeling, cancer biology, treatment planning software, validation, translational research

## Abstract

**Introduction:**

CytoReductive Surgery (CRS) followed by Hyperthermic IntraPeritoneal Chemotherapy (HIPEC) is an often used strategy in treating patients diagnosed with peritoneal metastasis (PM) originating from various origins such as gastric, colorectal and ovarian. During HIPEC treatments, a heated chemotherapeutic solution is circulated through the abdomen using several inflow and outflow catheters. Due to the complex geometry and large peritoneal volume, thermal heterogeneities can occur resulting in an unequal treatment of the peritoneal surface. This can increase the risk of recurrent disease after treatment. The OpenFoam-based treatment planning software that we developed can help understand and map these heterogeneities.

**Methods:**

In this study, we validated the thermal module of the treatment planning software with an anatomically correct 3D-printed phantom of a female peritoneum. This phantom is used in an experimental HIPEC setup in which we varied catheter positions, flow rate and inflow temperatures. In total, we considered 7 different cases. We measured the thermal distribution in 9 different regions with a total of 63 measurement points. The duration of the experiment was 30 minutes, with measurement intervals of 5 seconds.

**Results:**

Experimental data were compared to simulated thermal distributions to determine the accuracy of the software. The thermal distribution per region compared well with the simulated temperature ranges. For all cases, the absolute error was well below 0.5°C near steady-state situations and around 0.5°C, for the entire duration of the experiment.

**Discussion:**

Considering clinical data, an accuracy below 0.5°C is adequate to provide estimates of variations in local treatment temperatures and to help optimize HIPEC treatments.

## Introduction

Peritoneal metastasis (PM) originating from primary gastric, ovarian or colorectal cancer are often associated with poor survival (1–3). In general, we can identify three treatment strategies: systemic chemotherapy, surgery and cytoreductive surgery followed by Hyperthermic IntraPeritoneal Chemotherapy (HIPEC). Systemic chemotherapeutics are considered a palliative treatment strategy since the metastases on the peritoneal surface are highly hypoxic and are therefore difficult to treat systemically (4, 5). During cytoreductive surgery, the total tumor load can be significantly reduced resulting in an extension of patients’ life. However, microscopic nodules can remain on the peritoneal surface after surgery as well as circulating tumor cells in the peritoneal cavity, possibly limiting the long-term effect of the treatment. This residual disease can spread further across the peritoneal surface. To reduce this risk and to eliminate the microscopic disease, HIPEC can be administered directly after surgery. During HIPEC treatment, a heated chemotherapeutic solution (between 39-43°C) is circulated through the abdomen for a duration of up to 120 minutes. The chemotherapeutic agent is chosen based on the cancer origin and cytotoxic enhancement by heat. Before treatment, the chemotherapeutics are dissolved in a NaCl or Dextrose solution (6).

Although the biological and scientific rationale for the application of HIPEC is clear, HIPEC remains disputed because of varying clinical results. There are a limited number of randomized controlled trials investigating the combination of surgery and HIPEC and results vary. In 2018, *van Driel* et al. showed an increase of about 12 months in median overall survival for ovarian cancer patients suffering from PM treated with surgery and HIPEC compared to patients treated with surgery alone (7). However, in 2021 the PRODIGE-7 trial failed to show a survival benefit in colorectal cancer patients suffering from PM. Additionally, increased morbidity and toxicity was observed for patients in the HIPEC-arm (8). These mixed results can be interpreted as a reflection of the complexity of a HIPEC treatment in which the efficacy can be influenced by 8 different treatment parameters: patient selection, carrier solution, duration, delivery technique, perfusate volume, the choice of chemotherapeutics, dose and treatment temperature (6).

The perfusate used during HIPEC is heated to enhance the cytotoxic effect of the chemotherapeutics. This thermal enhancement depends strongly on the type of chemotherapeutic, cell type/cancer origin and locally reached temperature, quantified in the Thermal Enhancement Ratio (TER). The TER can be defined as the ratio of chemotherapeutic dose required to reach a specific endpoint at normothermic conditions (37°C) over the dose of chemotherapeutics required to reach the same endpoint at an enhanced temperature level (9). For *in vitro* studies, endpoints are usually defined in terms of cell survival. In an extensive *in vitro* study, *Helderman* et al. investigated a large array of chemotherapeutics and temperature combinations with a focus on different colorectal cell lines of various consensus molecular sub-types (CMS) (10). Chemotherapeutics considered were cisplatin, oxaliplatin, carboplatin, mitomycin-C (MMC), and 5-fluorouracil (5-FU) at 37-43°C with a 1 degree resolution. The two latter chemotherapeutics (MMC and 5-FU) did not show a direct synergistic effect with heat, while the platinum-based chemotherapeutics did, with TERs varying from 1.0 to 7.2 at 43°C, demonstrating a large variation among cell types. Focusing on one individual cell line, the TER can increase from 1.0 to 1.7-3.3 (oxaliplatin), 1.7-6.0 (cisplatin) and 1.4-7.2 (carboplatin) within the 37-43°C range, demonstrating the influence of temperature on the TER. To complicate things even more, not every TER-curve has a similar shape. For example, thermal enhancement can increase linearly with temperature. However, it is also possible that the TER increases rapidly after exceeding a certain threshold temperature within the 37-43°C range. This threshold is again dependent on type of chemotherapeutic agent and cell line. A complete data set of all possible combinations could be used for optimization of treatment strategies by choosing optimal thermal and chemotherapeutic conditions based on cancer cell characteristics. When optimal treatment conditions are determined, the next challenge is to realize these conditions in the entire target region during a HIPEC treatment. To achieve this, control over the thermal distribution is required which is difficult due to the large treatment volume and complicated dynamics and interactions. To represent intraperitoneal thermal distributions, studies often report on just inflow and outflow temperatures (11, 12) or measurements at one (unknown) abdominal site. The assumption is that when thermal losses between inflow and outflow are minimal or when a critical temperature is reached, the thermal distribution is stable enough such that chemotherapeutics can be administered. However, studies providing measurements at several locations have shown that this temperature monitoring approach does not provide an accurate representation of the actual thermal distribution, with possible variations between anatomical sites and patients of up to 3-4°C (13, 14). Given the often strongly temperature-specific TER, this can result in a very heterogeneous treatment of the peritoneal surfaces.

To ensure optimal HIPEC treatments these fluctuations should be prevented by adequate positioning of inflow and outflow catheters and optimizing flow patterns. Numerical simulations can be supportive to determine the most suitable treatment set-up and to this end, we developed treatment planning software, based on the computational fluid dynamics (CFD) OpenFoam software package (15). The software can predict flow patterns and provide insights into the spatial and temporal variations present in the peritoneal cavity during HIPEC. In previous work we used the software to optimize the preclinical HIPEC setup for rats (16). First, we performed simulations to find the optimal setup, realizing the most homogeneous thermal distribution in the peritoneum, after which we compared this setup with a setup that is standard during preclinical HIPEC treatments (16). Then, we experimentally treated rats using a standard and optimized setup, comparing the data with simulated thermal distributions (17). The experiments confirmed that the optimized catheter setup resulted in a more stable and homogeneous thermal distribution compared to the standard setup. The rat peritoneal cavity was divided into 4 quadrants and thermal losses were around 0.4°C lower for the optimized setup in all quadrants, demonstrating the usefulness of treatment planning software. Further steps towards human applications are ongoing. Since the peritoneal cavity of a human is larger and much more complicated, extensive experimental work is required to assess the accuracy of the predictive value of the software. The treatment planning software is based on two modules for calculating the drug and thermal distributions in the patient during HIPEC. Both distributions are crucial in determining local treatment conditions. In a previous study we validated the accuracy of the software for natural convection inside the hyperthermic range to be below 0.2°C (18). However, during HIPEC forced convection determines the flow pattern over an extensive and complex geometry.

This study focuses on further validation of the thermal module, accounting for forced convection during HIPEC. During a HIPEC treatment, large thermal gradients can occur resulting in thermal variations across the peritoneal surface. These gradients occur due to the large volume of the human peritoneal cavity and complex thermal interactions with surrounding tissues, vascular structures and the environment. To replicate these conditions we designed a phantom that can mimic clinical conditions for a HIPEC treatment. Several catheter setups, inflow temperatures and flow rates are considered and measurements are compared to simulations to determine the accuracy of the treatment planning software.

## Materials and methods

We subdivide this section into two parts. First, the experimental part of the study is discussed. Then the computational methods are discussed.

### Experimental

The aim of this study was to develop an experimental setup that is representative of a clinical HIPEC treatment. More specifically, for a thorough validation experiment, the observed thermal distribution should be comparable to the distribution occurring in the peritoneum during HIPEC. To achieve this, we developed a life-sized anatomically correct phantom representing the human peritoneum which can be used in a wide range of experimental conditions relevant for HIPEC. In the next sections we discuss the phantom design, experimental setup and measurement method.

#### Phantom creation

**Figure 1:**
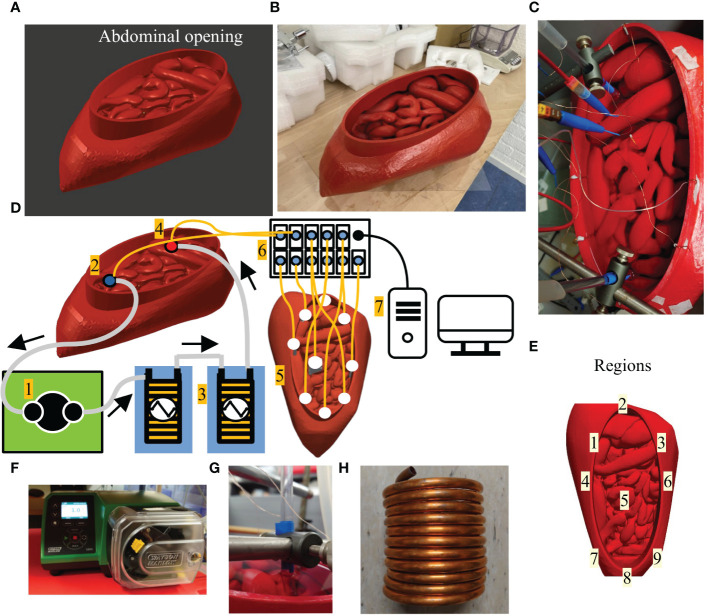
The 3D design of the phantom **(A)** and the 3D-printed phantom based on the design in A **(B)**. The oval opening of the peritoneal cavity (see label abdominal opening) mimics a “Colosseum” setup, often used during an open HIPEC treatment. Panel **(C)** shows a photograph of the phantom during experimentation. Schematic diagram of the setup used during experiments **(D)**. A roller pump (1) circulates water from the outflow (2) through two heat exchangers (3) placed inside water baths and back into the phantom (4). Temperatures are measured in 9 different regions (5) using 9 7-point thermocouple probes. The thermometry system (6) records temperatures which are monitored and stored on a PC (7). In panel **(E)** we visualize the location of the various probe regions used for evaluation of thermal profiles. Panels **(F–H)** show photographs of the roller pump, catheter tips locked in position and heat exchanger, respectively.

The phantom was based on the 4D extended cardiac-torso (XCAT) phantoms, developed by the Duke University. These models provide high resolution segmented anatomical data sets, based on segmentations of patients and the Visible Male and Female anatomical datasets from the National Library of Medicine (19). The volume of the organ models that can be generated using these phantoms are representative of 50th percentile males and females, based on height and weight (20) For the creation of our phantom we chose the female model because of the peritoneal extension in the pouch of Douglas, resulting in a more complex model. The organ models were imported as delineations into 3D slicer (21, 22) to create a peritoneal surface. HIPEC treatments can be performed with an opened or closed abdomen, each with their respective (dis)advantages. For this study we chose to design a phantom based on an open HIPEC treatment, since larger thermal gradients are expected during open HIPEC treatments compared to closed HIPEC treatments, which will thus be a better test for the model performance. All surfaces were imported into the 3D modelling and rendering package Blender (23) to create a 3D-printable model, see **Figure 1A**. The model was printed in two different parts using a Fortus 450mc 3D-printer (*Stratasys*). Walls consisted of 4 layers of acrylonitrile styrene acrylate (ASA) red (*Stratasys*), all 0.508 mm thick. After printing, the two parts were connected using mortise and tenon connections. The outside of the model was covered with a PVC coating to make the phantom waterproof. Organs and peritoneal exterior were not coated to allow water to seep in, filling the organs with water. This was done to mimic tissues and generate a realistic thermal conductivity. It took about one day to fill the organs and peritoneal exterior with water and therefore, the phantom set-up was stabilized at the start of the experiments and no additional water was seeping into the organs during experiments. An additional effect was the thermal interaction between the relatively cold organs and relatively warm peritoneal cavity which also occurs during HIPEC treatments. The 3D-printed phantom is shown in **Figure 1B**.

#### Experimental setup

The phantom described in the previous section was used in the experimental setup, shown schematically in **Figure 1C**. For clarity we photographed several key elements of the setup in **Figures 1D–H**. The phantom was filled with water, in total around 2.6 L. A roller pump (Label 1) was used (WatsonMarlow 530S/R2, Falmouth, United Kingdom) to circulate the fluid from the outflow (Label 2) into two heat exchangers (Label 3) placed in separate water baths(Lauda aqualine AL12, Beun–De Ronde BV, Abcoude, the Netherlands) and then on to the inflow (Label 4). The heat exchangers consisted of two hollow copper coils through which the water was able to flow. The two coils were connected in series to ensure rapid heating and a stable inflow temperature. Both inflow and outflow temperatures were monitored. In the phantom we placed 9 multi-sensor type T thermocouple probes (Ella-CS, Hradec Kralove, Czech Republic), each having 7 measurement points, separated by1 centimeter with an accuracy of 0.01-0.1°C and an accuracy < 0.1°C. The probe locations are shown at Label 5. The thermocouple probes were placed such that the temperature was measured over a length of 7 centimeter on each location. In total, the temperature was measured at 7 × 9 = 63 locations in the phantom. In this way, we were able to capture an adequate representation of the thermal gradients in that region. The temperature was measured by a 196 channel thermometry system (Label 6) and monitored on a computer (Label 7). The thermocouples were linearly calibrated between 25-45°C using an Isotech TTI-10 thermometer with a probe (Isotech 935–14–61) that has an accuracy < 0.05°C. For some setups, the inflow tube was split into several inflow catheters. The radius of the circular inflow and outflow catheters was 3.5 mm and featured one single hole at the end of the catheter. The tips of the catheters were solid and fixed into position (see **Figure 1D**) during the experiments to prevent changes in position and/or orientation to occur due to the pulsatile nature of the roller-pump.

#### Measurements and different treatment setups

The experimental setup was always allowed to cool down to room temperature to ensure the same baseline temperature in the entire phantom and its environment before experimentation started. This provided the required well-controlled uniform initial conditions for the simulations. Before experiments started, air bubbles were removed from the system by performing a pre-circulation for 10 minutes. The circulation was stopped and water baths were turned on and set to 43.5°C, resulting in an inflow temperature of about 42.7°C. When temperatures in the water baths were stabilized, measurements were started with an interval of 5 seconds. Circulation was started again and continued for a total duration of 30 minutes after which all systems were turned off stopping measurements.

**Figure 2:**
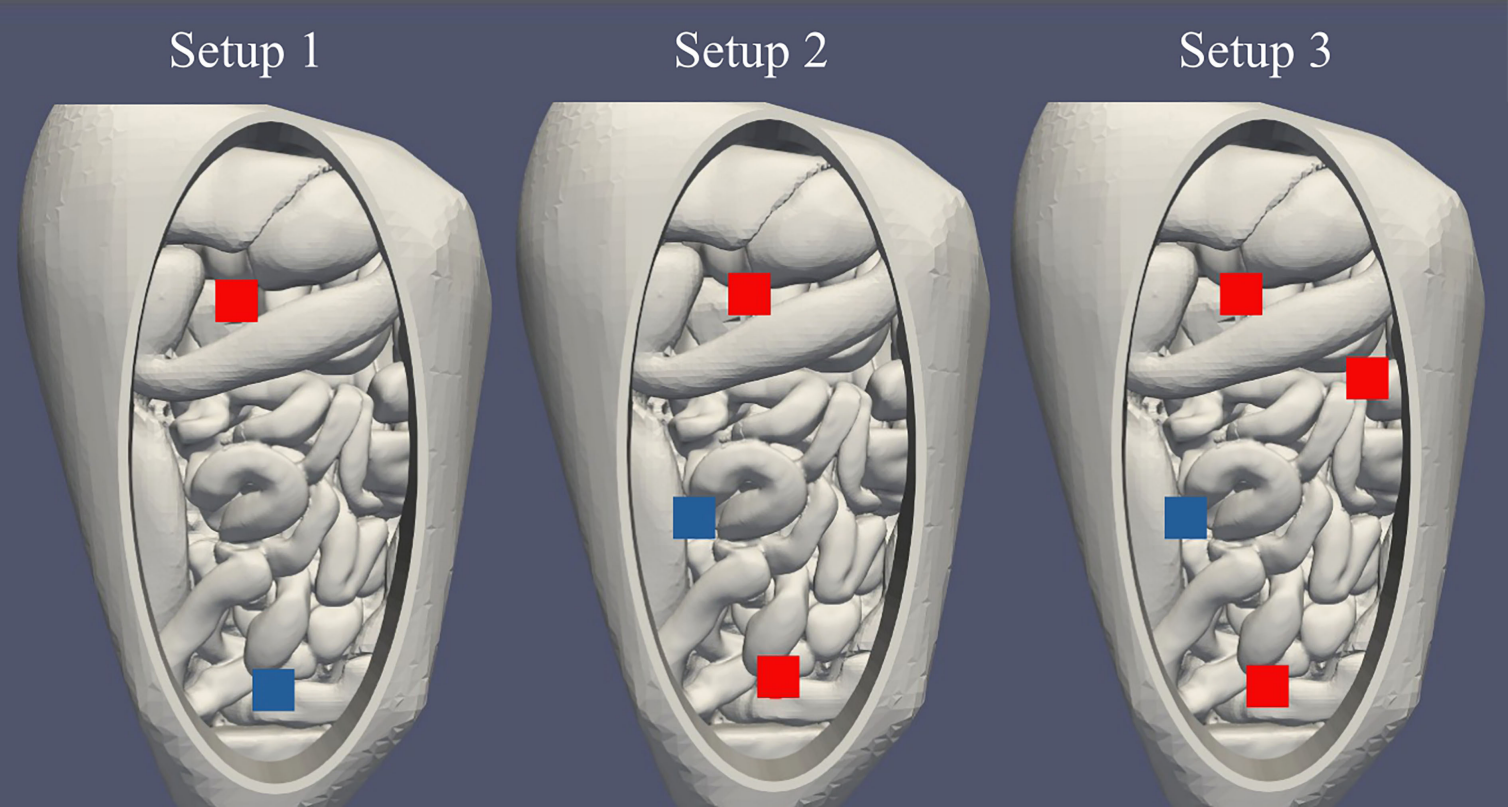
Visualization of the catheter setups used in this study. From left to right we show the positions for the 1 inflow/1 outflow, 2 inflow/1 outflow and 3 inflow/1 outflow cases, respectively. Inflows are visualized in red, the outflow in blue. For setup 1, the inflow was positioned near the liver and the outflow was positioned near the rectum. For setup 2, inflows were positioned near the liver and rectum and the outflow was positioned slightly right to the patient’s center to allow the catheter tips to be at a depth of 3 cm. For setup 3, inflows were positioned near the liver, descending colon and rectum and the outflow was positioned slightly right to the patient’s center.

In total, 7 cases were investigated, considering changes in catheter setup and flow rates. As a baseline case, the 1 inflow and 1 outflow catheter setup was used at a flow rate of 1000 mL/min. We repeated this experiment 3 times to demonstrate the reproducibility of the experiments. Using the same catheter setup, 3 different flow rates were considered: 600 mL/min, 800 mL/min and 1000 mL/min. For the base flow rate of 1000 mL/min, 3 catheter setups were considered: 1 inflow/1 outflow, 2 inflow/1 outflow and 3 inflow/1 outflow. The outflow catheter was placed at a maximum distance from the inflow catheter(s), which can be considered optimal since this positioning allows the heat to distribute before extracting it. Only additional inflow catheters were considered because additional outflow catheters would not have impacted the thermal distribution as significantly as additional inflow catheters. The 1 inflow/1 outflow setup with a flow rate of 1000 mL/min setup was also used for with inflow temperatures of 37.7°C and 47.7°C. **Figure 2** shows the various catheter setups and **Table 1** provides an overview of the case descriptions. Since the depth of the catheter tips can influence flow patterns, catheter tips were placed at a fixed depth of 3 centimeter from the fluid surface, resulting in controlled inflow conditions for the simulations.

**Table 1:** Catheter setup and flow rates considered in this study.

Case	Setup	Flow rate [mL/min]	Inflow temp. [°C]	Description
#1	1	1000	42.7	Baseline case repeated 3 times to demonstrate reproducibility. Inflow positioned near liver. Outflow positioned near rectum.
#2	1	800	42.7	Reduced flow rate to increase gradients and lower overall temperature.
#3	1	600	42.7	Reduced flow rate to increase gradients and lower overall temperature.
#4	1	1000	37.7	Reduced inflow temperature to lower overall temperature.
#5	1	1000	47.7	Increased inflow temperature to increase overall temperature.
#6	2	1000	42.7	Inflows positioned near liver and rectum. Outflow positioned centrally.
#7	3	1000	42.7	Inflows positioned near liver decending colon and rectum.Outflow positioned centrally.

Setups are visualized in **Figure 2**.

### Simulations

For the simulations, we used the treatment planning software that we have developed for HIPEC (192 24). The software was based on the OpenFoam software package (15). In 193 this section we discuss the numerical methods, computational geometry and corresponding boundary 194 conditions.

#### Numerical methods

In creating the treatment planning software, the OpenFoam *chtMultiRegionFoam* solver was extended to incorporate biological processes regarding thermal and drug dynamics (24). For this particular study, these processes are absent and therefore, the software used can be considered to be the original *chtMultiRegionFoam* solver. The solver is based on a combination of the SIMPLE (Semi-Implicit Method for Pressure-Linked Equations) and the PISO (Pressure Implicit with Splitting of Operator) algorithms, referred to as PIMPLE (Pressure Implicit with Splitting of Operator). We employed several discretization schemes: Crank-Nicolson for time, cellLimited for finite volume and linearUpwind for all other relevant fields (temperature, velocity, pressure etc.).

The most fundamental dynamics can be described by three equations, governing the thermal dynamics and determining the flow patterns. These are the energy equation, momentum conservation and mass conservation equations, written as


(1) $$\frac{\partial \rho h}{\partial t}+\nabla \cdot \left(\rho \vec{U}h\right)+\frac{\partial \rho K_{e}}{\partial t}+\nabla \cdot \left(\rho \vec{U}K_{e}\right)-\frac{\partial p}{\partial t}=-\nabla \cdot \vec{q},$$

(2) $$\frac{\partial }{\partial t}\rho \vec{U}=-\nabla \cdot \left(\rho \vec{U}\times \vec{U}\right)-\nabla \cdot \tau -\nabla p+\rho \vec{g}$$

(3) $$\frac{\partial \rho }{\partial t}=-\nabla \cdot \left(\rho \vec{U}\right)$$

respectively. In Equation (1), (2) and (3) t, $\vec{q}$ , *h* , *K*_*e*_ , $\vec{U},\rho ,\tau ,p,\vec{g}$ are time, the heat flux [*W*/*m* ^2^ ], enthalpy [*m* ^2^/*s* ^2^ ], specific kinetic energy [*m* ^2^/*s* ^2^ ], velocity [*m*/*s* ], density [*kg*/*m* ^3^ ], shear-rate tensor [*kg*/*m*/*s* ^2^ ], pressure [*Pa* ] and the gravitational vector [*m*/*s* ^2^ ], respectively. For a more complete and detailed description we refer to (25).

#### Computational geometry

**Figure 3:**
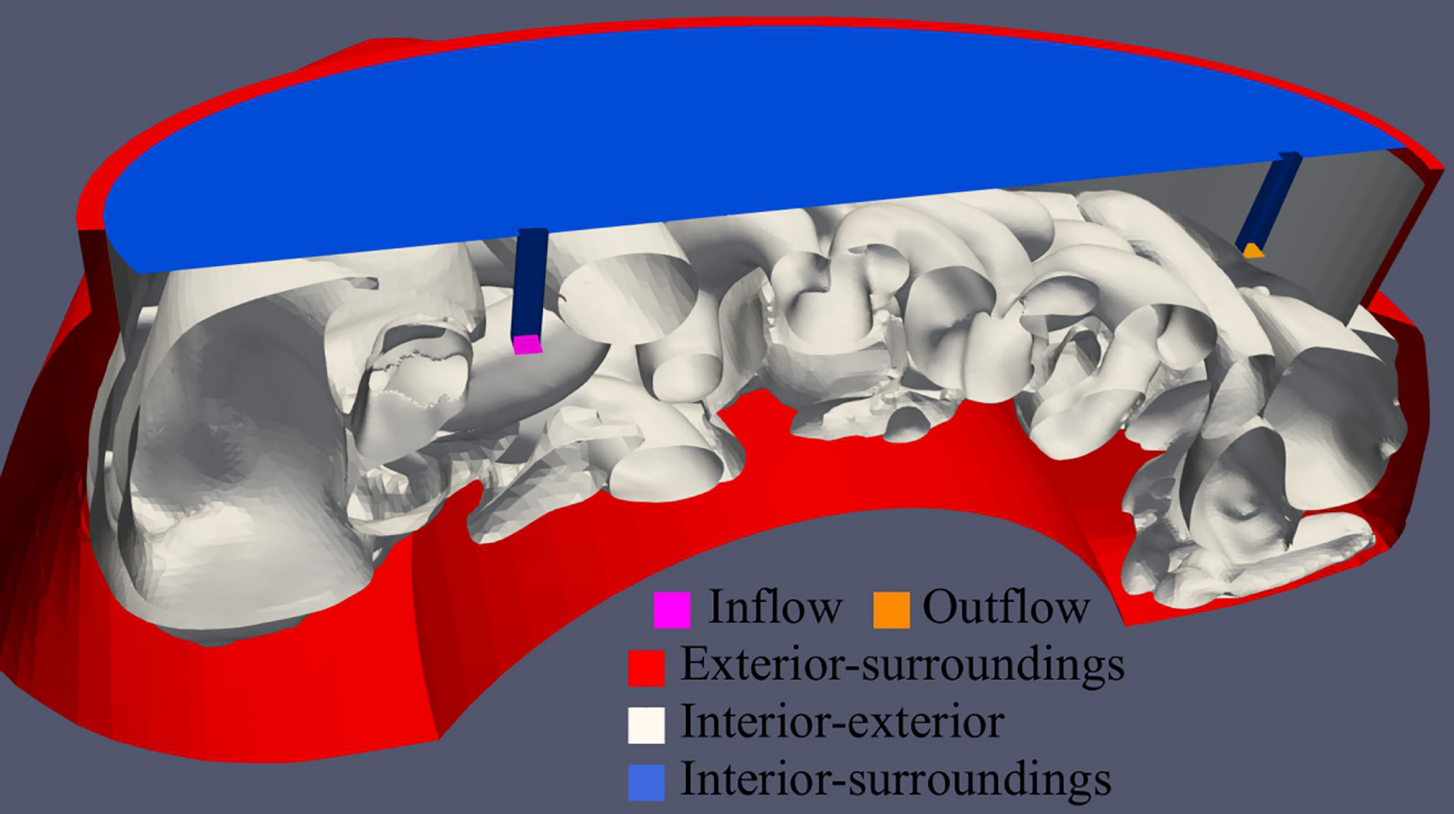
Visualization of the computational geometry used during simulations. In total, 5 boundaries were defined: inflow and outflow patch (pink and orange, respectively), peritoneal exterior to the surroundings (red), peritoneal interior to the peritoneal exterior (white) and the peritoneal interior to the surroundings (blue).

For the design of the computational geometry, we used the same computer-aided design (CAD) stereolithography (.stl) files used for the design of the phantom resulting in a 1-to-1 correspondence between phantom and computational geometry. This region constituting the inside of the phantom was defined as peritoneal exterior. Since fluid in this region is stationary, this region was modelled as a solid with a heat conductivity equal to water. The peritoneal interior was designed to incorporate the various catheter setups. The inflow and outflow catheters were modeled as square tubes instead of circular tubes to reduce complexity and computational time. Circular catheters require significantly smaller elements to accurately represent the surface area of the catheter opening. The time-step is determined by the Courant number, calculated by the ratio of the velocity, times-step and element-size $C=\frac{u\cdot \Delta t}{\Delta x}<1$ . The time-step is a fixed parameter but the velocity and element size vary. Since the velocity is largest near the catheters, it is crucial to keep element sizes large enough to allow sufficiently large time-steps to reduce computational times without losing accuracy. To preserve flow characteristics, the total inflow surface area of the square tubes was equal to the inflow surface area of the circular catheters during experimentation. The 3.5 mm radius with a flow rate of 1000 mL/min resulted in a (maximal) Reynolds number of ≈3000 near the catheters, quickly decreasing away from the catheters. To account for turbulence, we applied the *k*−*ω* model with wall functions that can be used for high and low Reynolds numbers. Meshes used typically consisted of 95000-97000 hexahedral elements, depending on the number of catheters. Mesh elements varied in size, with a minimal volume in the order of 0.1 *mm* ^3^ to represent the complex regions in the geometry. Larger fluid volumes were represented by larger elements to reduce computational times. In total, this resulted in 5 boundaries: between interior and exterior of the peritoneal cavity, from the peritoneal exterior to the surroundings, from the peritoneal interior to the exterior, inflow patch(es) and outflow patch. These are visualized in **Figure 3**.

#### Boundary conditions

For these simulations, 5 fields are relevant: pressure, velocity, temperature and the turbulence conditions k and *ω* . Here we discuss the different boundary conditions used during simulation and in **Table 2** we list which boundary conditions is used for which boundary and field. The boundary condition *fixedValue* prescribes the value at the boundary. The *zero-Gradient* conditions set the normal gradient at the boundary to zero such that the boundary value equals the value of the neighboring cell. The *inletOutlet* condition is a combination of both. In case of a negative flux, the condition is considered *zero-Gradient* and for reverse flow, the value is fixed to a prescribed value. For turbulence conditions for *k* and *ω* we used *turbulentIntestityKineticEnergyInlet*, *turbulentMixingLengthFrequencyInlet*, *kLowReWallFunction* and *omegaWallFunction* providing methods for calculating and initiating the kinetic turbulent energy and dissipation rate of the turbulent kinetic energy. For a full overview of all boundary conditions available in *OpenFoam* we refer to (26).

**Table 2:** Boundary conditions used in this study.

	Inflow	Outflow	Interior-exterior
**U**	*Pulsatile*	*Pulsatile*	*fixedValue (0,0,0)*
**p**	*zero-Gradient*	*fixedValue (0)*	*zero-Gradient*
**k**	*turbulentIntensityKineticEnergyInlet*	*inletOutlet*	*kLowReWallFunction*
*ω*	*turbulentMixingLengthFrequencyInlet*	*inletOutlet*	*omegaWallFunction*
**T**	*Specified by experimental conditions*	*inletOutlet*	*turbulentTemperatureCoupledBaffleMixed*
	Interior-surroundings	Exterior-surroundings	
**U**	fixedValue (0,0,0)	NA
**p**	zero-Gradient	fixedValue (0)
**k**	kLowReWallFunction	NA
*ω*	omegaWallFunction	NA
**T**	externalWallHeatFluxTemperature	externalWallHeatFluxTemperature

The “*Pulsatile*” boundary condition used for the velocity field *U* was designed to mimic the pulsatile behavior of the roller pump that was used during experimentation. We define the inflow condition as


(4) $$U_{i}=\left(0,-A\sin\;2\pi tf+B,0\right),$$

where *A* , *t* , *f* and *B* are the amplitude [*m*/*s* ], time [*s* ], frequency [1/*s* ] and average velocity [*m*/*s* ], respectively. The outflow condition is exactly the same, but in opposite direction.

The thermal boundary condition between peritoneal interior and exterior calculates the heat transfer based on the conductivity on either side of the boundary, slowed down by the thermal resistance generated by the material in between. The thermal resistance is determined by the materials thickness (*d*) and the thermal conductivity (*k* ) of the material:


(5) $$R_{th}=\overset{n}{\underset{i=1}{\sum }}\frac{d_{i}}{k_{i}}.$$

For the ASA material used in our phantom, this was 4×0.508*mm*=2.32*mm* and *k*=0.16*W*/*m* , respectively. The thermal resistance is used on both sides to calculate the temperature and gradient at the boundary such that patches on both sides have equal temperature and gradients.

The boundary between the peritoneal interior and surroundings involves the heat exchange between fluid surface and surrounding air. This transfer is reflected in the total heat transfer coefficient *h* [*W*/*m* ^2^/*K* ]. This variable can vary substantially (~10−100*W*/*m* ^2^/*K* ). We chose a value corresponding to a value that was used in a previous study, regarding the interaction in a rat model (*q*=660*W*/*m*2 ). This resulted in a value of *h*≈30*W*/*m* ^2^/*K* equalling 600*W*/*m* ^2^ in case of a 20 degree temperature difference. This condition was also used for the boundary between peritoneal exterior and surroundings including an additional thermal resistance in the form of print material.

#### Evaluation

Simulated thermal profiles were evaluated in a small volume around the location of the probe to account for position uncertainties. Not all probe volumes were of similar size and depth. Some probes had to follow the phantoms local curvature. For example, probe 2 and 4 had to curve around the top of the liver and ascending colon, respectively. Probe 1, 3 and 6 could be oriented directly downward and are therefore placed deeper. This directly affected the probe volume. When possible, the evaluation volumes around the probes were around 1 cm in each direction. For some regions, the geometry did not allow 1 cm in each direction and therefore the evaluation volume was smaller. In each probe volume, the average, minimum and maximum simulated temperatures were stored with an interval of 60 seconds for a duration of 1800 seconds. These values were compared to experimental values obtained according to the “Experimental” section. Over the 1800-second time frame, we distinguished between the “temporal” behavior evaluated over the entire duration and the “steady-state” behavior, evaluated over the last 5 minutes.

## Results

This section is subdivided into four parts relating to first the baseline case followed by three different objectives regarding the treatment planning software. First we will show the experimental results per probe for the 1 inflow/1 outflow case using 1000 mL/min and compare measurements with simulations and determine the amount of uncertainty that can be expected from the experiments. This serves as the baseline case, next we will evaluate the effect of flow rate, inflow temperature and catheter setup on the thermal distribution.

### Baseline case (case #1)

**Figure 4:**
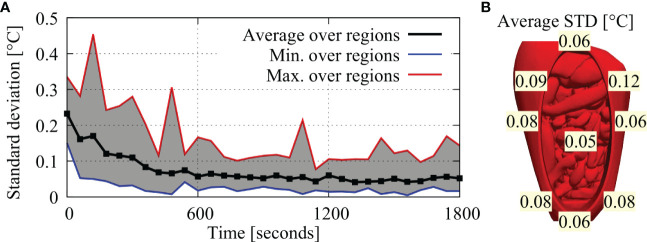
**(A)** Minimum, maximum and average standard deviations over all regions, plotted over time. Standard deviations are highest near the start of the experiment and decrease towards a steady-state near the end of the experiment. **(B)** Average standard deviations per region.

In this section we present the results for the experiments with the baseline setup, which we repeated 3 times. The steady state (last 5 minutes) temperatures are shown in the first three columns of **Table 3**. All regions are comparable over the three repetitions with most temperatures within one tenths of a degree. This is also reflected in the standard deviation being typically lower than 0.1°C. The standard deviations over the full duration of the experiment are also plotted over time in **Figure 4A** showing a decrease towards a steady state around 0.05°C on average. In **Figure 4B**, we report the average standard deviation per region, which was also below 0.1°C. The maximum values were observed in the first ten minutes for regions 1, 3 and 4, which are located closest to the inflow catheter and are therefore subject to high thermal gradients.

**Table 3:** Steady state temperatures and standard deviations baseline case.

	Steady state temperatures and standard deviations baseline case			
	Experiment 1	Experiment 2	Experiment 3	STD Ave.
Region 1	40.99	40.88	40.89	0.09
Region 2	39.58	39.65	39.6	0.04
Region 3	40.02	40.06	40.04	0.10
Region 4	41.48	41.54	41.51	0.03
Region 5	41.43	41.49	41.46	0.03
Region 6	39.46	39.52	39.49	0.04
Region 7	40.73	40.78	40.75	0.04
Region 8	40.66	40.7	40.69	0.03
Region 9	40.49	40.6	40.53	0.07
Outflow	41.16	41.22	41.21	0.04

Column 1, 2 and 3 show the average steady state temperature in the last 5 minutes for three repetitions of the baseline case experiment (1 inflow, 1 outflow at 1000 mL/min). Column 4 shows the average standard deviations.

**Figure 5:**
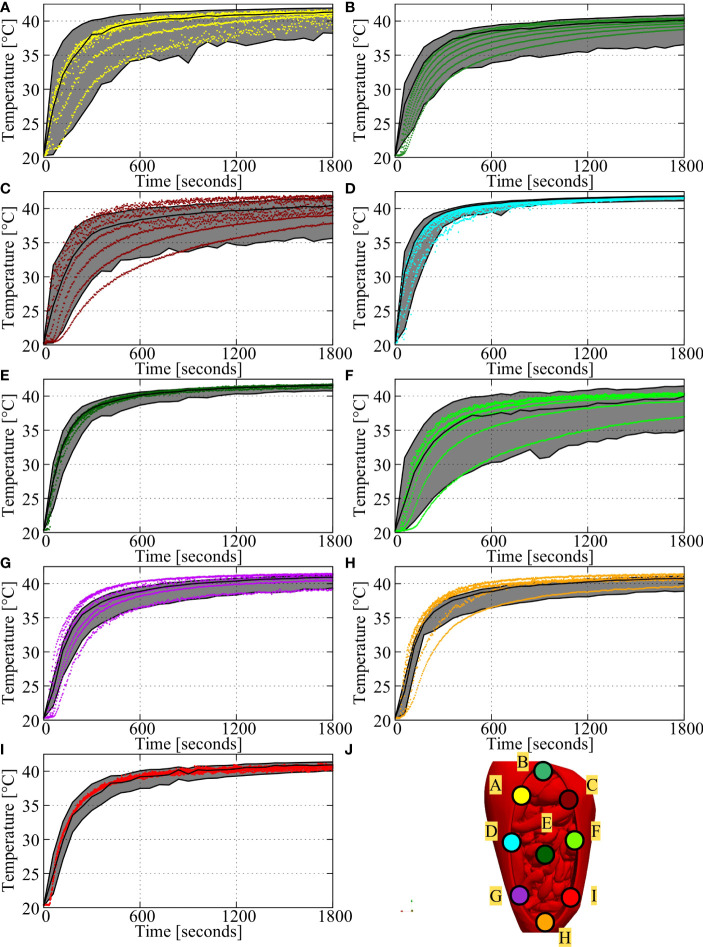
Comparison of measurements and predicted thermal ranges for the baseline setup (case # 1). **(A-I)** shows the comparison for region 1-9, with the locations of these regions shown in **(J)**. Simulated averages and ranges are represented by the black lines and shaded areas and measurements are represented by the colored dots. Measurements were taken every 5 seconds, with probes featuring 7 measurement points per probe. Therefore, each plot shows 2520 measurements over the duration of the experiment. In general, measurements fall within the predicted ranges, especially near steady state (last 5 minutes).

In **Figure 5** we compare the simulated thermal profiles and measured values per probe location. Panel A-I show the results for region 1-9 with panel J showing the location of each probe. In general, simulations were able to predict the measured ranges accurately providing correct estimates on which region would have a higher amount of variation. For example, regions 1, 3 and 6 (panels A, C and F) were predicted to have a large difference between minimum and maximum values, which was confirmed by the experiments. Regions 4, 5 and 9 were predicted to be relatively homogeneous, again confirmed by experiment. For regions 3, 7 and 8 (panels C, G and H), some measurements within the first 10 minutes of the experiment were outside the predicted range. Temporal gradients are initially quite high making it harder to predict and to measure the temperature resulting in relatively large uncertainties in the first part of the experiment. This is also reflected by **Figure 4**, showing relatively large standard deviations in the first 10 minutes. Furthermore, the presence of the thermal probes could have impacted the measurements by occasionally blocking the flow in narrow regions (regions 7 and 8), impacting one or two points on the probe.

### Velocity variation (cases #1, #2 and #3)

Next, the flow rate for the 1 inflow and 1 outflow catheter setup was varied from 600 mL/min to 1000 mL/min. Increasing or decreasing flow rates directly impacts the total amount of heat that is delivered to the peritoneal cavity and is therefore an important variable to optimize. Besides the amount of heat delivered to the system, a higher flow rate can result in an earlier onset of equilibrium. On average, the onset of a Δ*T*<0.1 °C per minute was 5-6 minutes earlier for 1000 mL/min compared to 600 mL/min, for both simulated and measured values.

**Figure 6:**
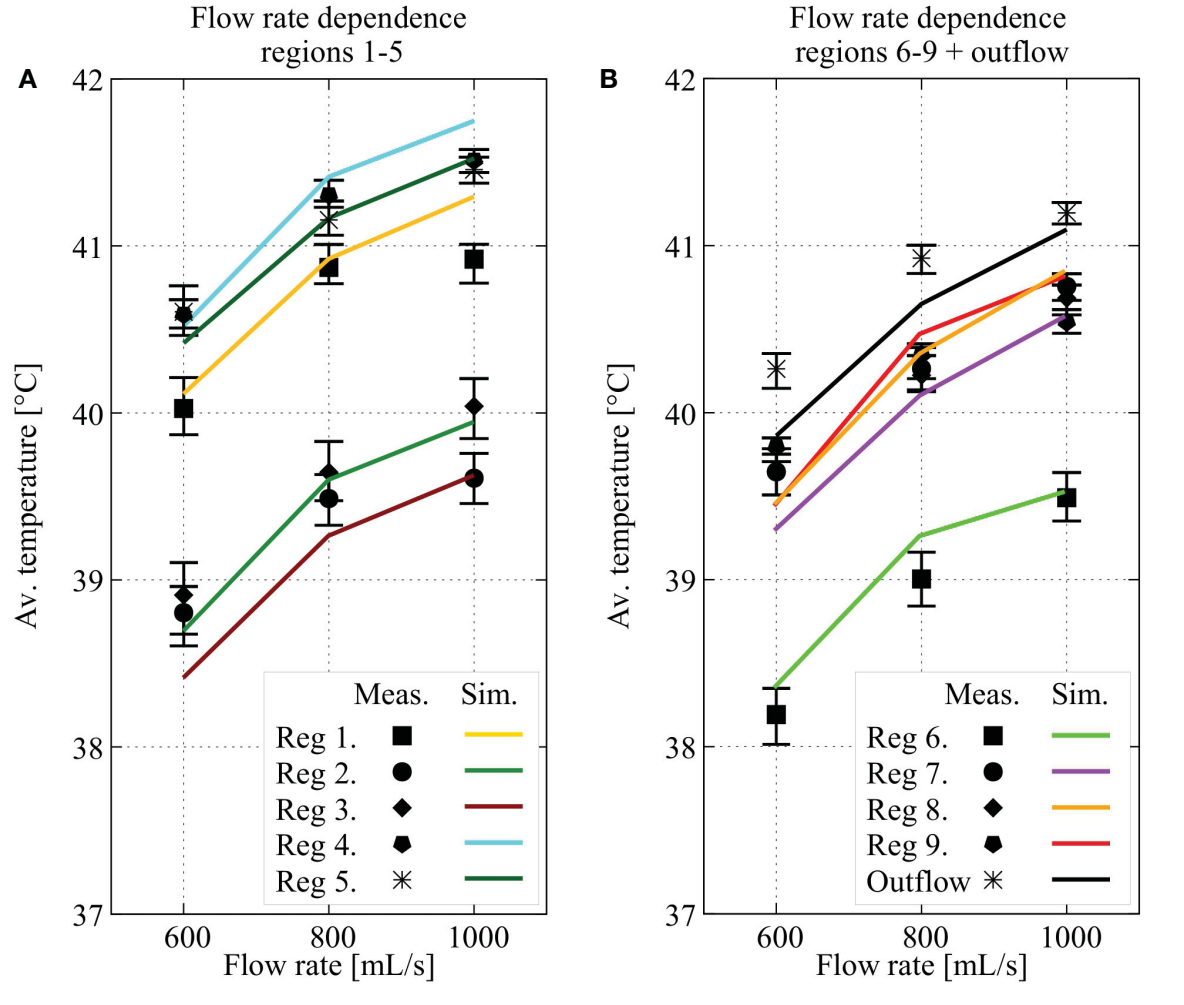
Effect of the flow rate on the temperature in each region and on the outflow temperature. In figure **(A, B)**, average values for region 1 through 5 and 6 through 9 plus outflow are plotted for three different flow rates (600, 800 and 1000 mL/min),respectively. The values shown are calculated as the average over the last 5 minutes of the predicted (colored line) or measured values (min/max values in that last five minutes shown by the whiskers and the average shown by the symbols). All regions show an increase in treatment temperature after 25 minutes, correctly predicted by simulated profiles.

**Figure 7:**
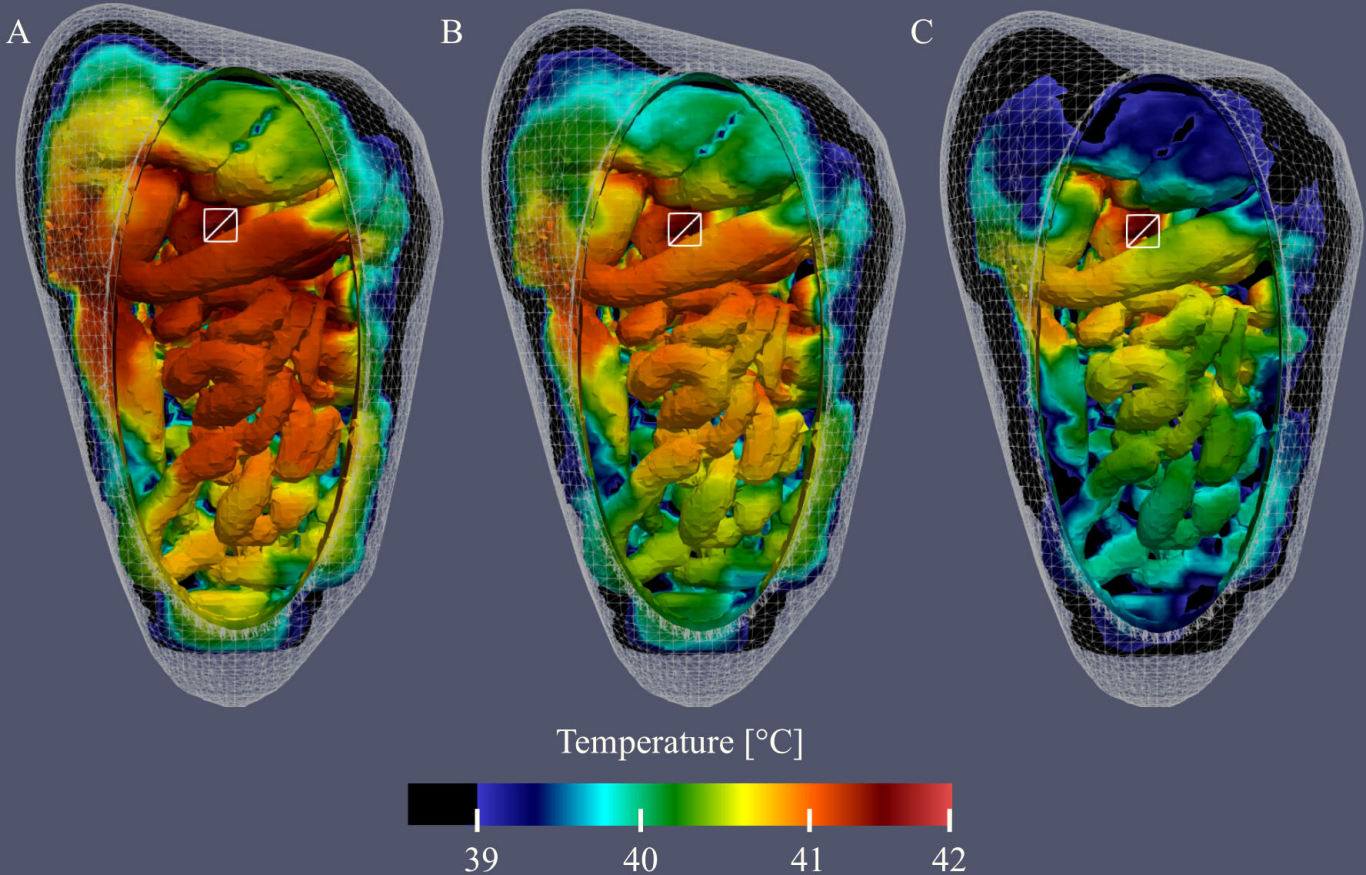
Visualization of the simulated thermal distributions and the effect of changes in flow rate on the distribution. White squares denote the location of the inflow catheter and temperatures below 39°C are black. The thermal distribution of case #1 **(A)** shows that a high flow rate at one location results in high temperatures near the inflow and surroundings. Reducing the flow rate to 800 mL/min **(B)** and 600 mL/min **(C)** results in lower temperatures, most notably in distant regions.

The high(er) flow rates resulted in increased local temperatures for all regions. In **Figure 6**, we show the average, minimal and maximal measured temperature over the last 5 minutes by the symbols and whiskers, respectively. The simulated temperatures are represented by the colored lines. A similar relationship can be seen for all regions, accurately predicted by the simulated values. The increase in temperature is larger comparing 600 to 800 mL/min than it is when comparing 800 mL/min and 1000 mL/min suggesting a rapid increase in temperature for variations at relatively low flow rates and approaching a plateau for variations at higher flow rates. Regions 1 and 3 were observed to have the largest error between simulation and experiment. The probes in these two regions were not curved behind the phantom geometry and were placed directly down close to the inflow. The flow pulsation resulted in slightly sinusoidal temperature profiles for some points on these two probes. Temperatures increased at maximum velocity, after which temperatures leveled again at minimal velocity. The sinusoidal pulsatile simulation condition was used to account for this pulsatile flow behavior. However, a small phase shift between the experimental and simulated inflow conditions could result in relatively large errors. The increase in treatment temperatures for higher flow rates is also visualized in **Figures 7A–C**. **Figure 7A** shows that the high flow rate case achieves high temperatures near the inflow catheter. For lower flow rates, the reach of the heat decreases, significantly impacting temperatures in distant regions.

In **Table 4** we listed the absolute errors for the flow rate variations. The first three columns show the absolute errors averaged over the last 5 minutes and the last three columns show the averaged absolute error over the entire treatment duration. Absolute errors below 0.5°C are made bold. Absolute errors in the last 5 minutes were below 0.5°C for all regions and outflow. On average, the absolute errors were below 0.25°C. Averaging the absolute error over the entire duration, errors exceeded 0.5°C for regions 1 (600 & 1000 mL/min), 2 (600, 800 & 1000 mL/min), 3 (800 & 1000 mL/min), 4 (1000 mL/min) and 6 (600 & 1000 mL/min). These averages are predominantly caused by large(r) errors in the first part of the experiment. More specifically, 86% of the errors larger than 0.5°C were observed in the first half of the experiment.

**Table 4:** Absolute errors between predicted an measured values for flow rate variations (cases #1, #2 and #3).

	Steady state			Overall		
Flow rate [mL/min]	600	800	1000	600	800	1000
Outflow [°C]	**0.40**	**0.21**	**0.10**	**0.43**	**0.30**	**0.27**
Region 1 [°C]	**0.12**	**0.05**	**0.37**	**0.40**	0.52	1.11
Region 2 [°C]	**0.11**	**0.11**	**0.33**	0.83	0.75	1.16
Region 3 [°C]	**0.50**	**0.38**	**0.41**	0.52	**0.46**	0.65
Region 4 [°C]	**0.10**	**0.11**	**0.24**	**0.23**	**0.40**	0.59
Region 5 [°C]	**0.19**	**0.02**	**0.07**	**0.23**	**0.14**	**0.12**
Region 6 [°C]	**0.16**	**0.26**	**0.08**	0.59	**0.48**	0.53
Region 7 [°C]	**0.20**	**0.09**	**0.09**	**0.22**	**0.32**	**0.20**
Region 8 [°C]	**0.35**	**0.12**	**0.11**	**0.39**	**0.29**	**0.31**
Region 9 [°C]	**0.35**	**0.12**	**0.27**	0.51	**0.32**	**0.24**
Average [°C]	**0.25**	**0.15**	**0.21**	**0.43**	**0.40**	0.52

Differences below 0.5°C are denoted in boldface. Near steady state, all values were below 0.5°C. Over the entire duration of the experiment, differences could exceed 0.5°C, mostly due to outliers during the first 10 minutes where temporal gradients were largest and measurement errors were highest, see **Figure 4**.

### Thermal variation (cases #1, #4, #5)

**Figure 8:**
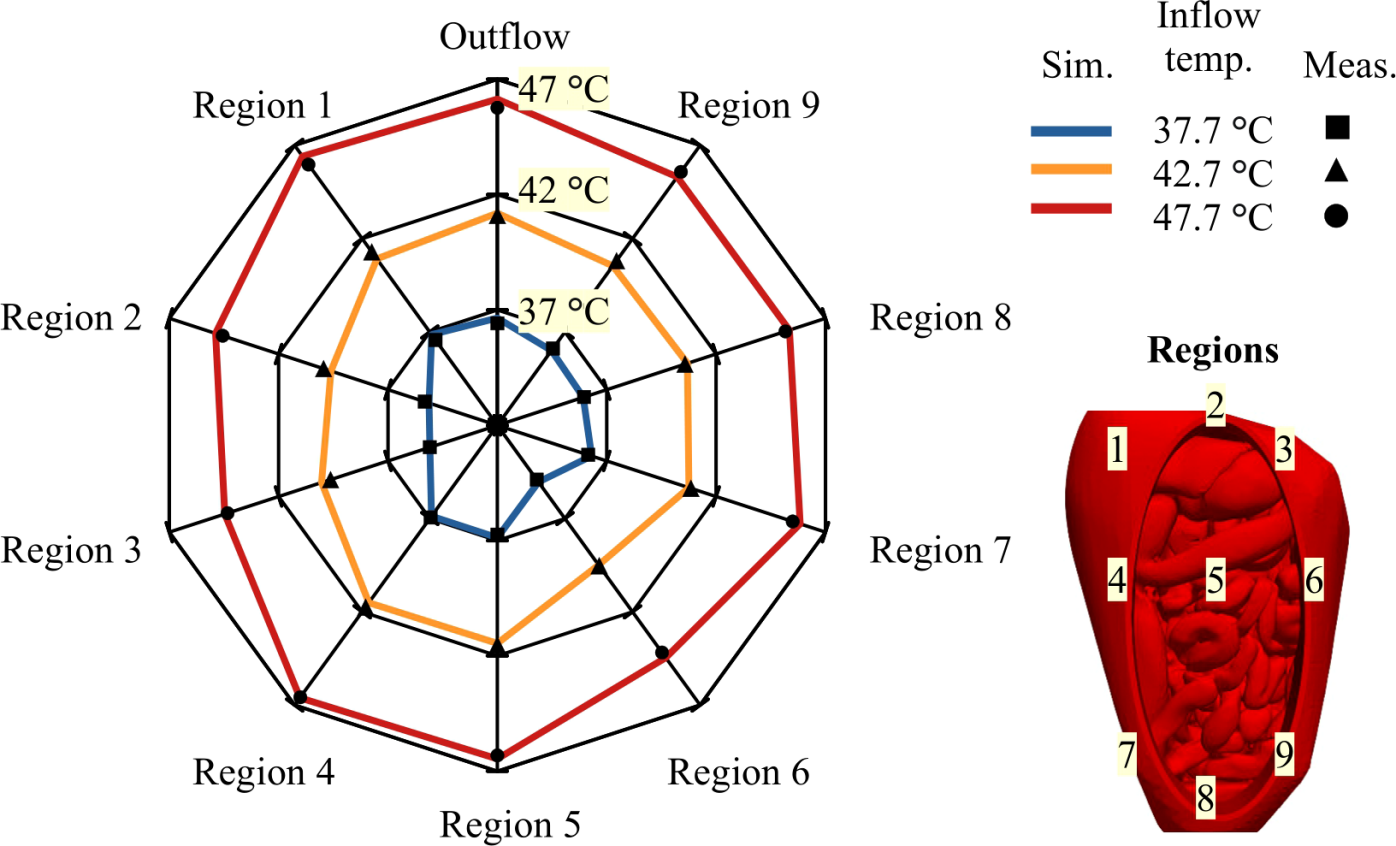
Web diagram showing the average steady state temperature for different inflow temperatures. Measured temperatures are visualized by a ■ , ▲ and • for inflow temperatures of 37.7°C, 42.7°C and 47.7°C, respectively. Simulated temperatures are represented by a blue, orange and red lines for inflow temperatures of 37.7°C, 42.7°C and 47.7°C, respectively. Similar distributions are visible for all inflow temperatures. Region 1, 4, 5 and the outflow are all close to the inflow temperature while region 2, 3 and 6 are well below the inflow temperature. Ideally, all temperatures would approach their respective upper levels, e.g. 37°C, 42 text degree C and 47°C.

The treatment planning software should be accurate for the entire hyperthemic temperature range (37-43°C). Therefore, we performed additional experiments with an inflow temperature 37.7°and 47.7°C with 1 inflow at 1000 mL/min. In **Figure 8** we show a comparison between the simulated and measured steady state temperatures for all nine regions and the outflow. Measured values (symbols) compare well with the simulated values (lines). In this diagram, we can distinguish three levels: 37, 42 and 47°C. In an optimal distribution, all regions would approach these limits. From **Figure 8** we can see that the relative spatial distribution is independent of the inflow temperature. For all inflow temperatures regions 1, 4, 5 and the outflow are close to the inflow temperature. Regions 2, 3 and 6 show large thermal losses compared to the inflow temperature. These variations are well predicted.

In **Table 5** we report the absolute difference for experiments performed with an inflow temperature of 37.7, 42.7 and 47.7°C. Columns 1-3 show the average absolute error during the steady state phase while columns 4-6 show the average absolute error over the entire duration of the experiment. During the steady state phase, all errors were below 0.5°C, with averages below 0.3°C. Considering the entire duration of the experiment, errors exceeded 0.5°C caused by large(r) errors in the first part of the experiment. Errors were larger for higher inflow temperatures. This was a result from larger gradients and variations that occur when treatment temperatures are higher. Overall, simulated values compared well with measured values.

**Table 5:** Absolute errors between predicted an measured values for varying inflow temperatures (cases #1, #4 and #5).

	Steady state			Overall		
Inflow Temp.	37.7°C	42.7°C	47.7°C	37.7°C	42.7°C	47.7°C
Outflow [°C]	**0.23**	**0.10**	**0.38**	**0.41**	**0.27**	0.81
Region 1 [°C]	**0.29**	**0.37**	**0.45**	**0.32**	1.11	0.59
Region 2 [°C]	**0.20**	**0.33**	**0.33**	0.58	1.16	0.88
Region 3 [°C]	**0.04**	**0.41**	**0.13**	0.57	0.65	0.61
Region 4 [°C]	**0.07**	**0.24**	**0.03**	**0.27**	0.59	**0.43**
Region 5 [°C]	**0.13**	**0.07**	**0.15**	**0.28**	**0.12**	0.62
Region 6 [°C]	**0.13**	**0.08**	**0.36**	**0.34**	0.53	0.82
Region 7 [°C]	**0.16**	**0.09**	**0.34**	**0.41**	**0.20**	0.70
Region 8 [°C]	**0.06**	**0.11**	**0.18**	**0.24**	**0.31**	0.56
Region 9 [°C]	**0.11**	**0.27**	**0.27**	**0.44**	**0.24**	**0.47**
Average [°C]	**0.14**	**0.21**	**0.26**	**0.39**	0.52	0.65

Differences below 0.5°C are denoted in boldface. Near steady state, all values were below 0.5°C. Including the entire range, differences could exceed 0.5°C, mostly due to outliers during the first 10 minutes where temporal gradients were largest and measurement errors were highest, see **Figure 4**.

### Catheter variation (cases #1, #6, #7)

**Figure 9:**
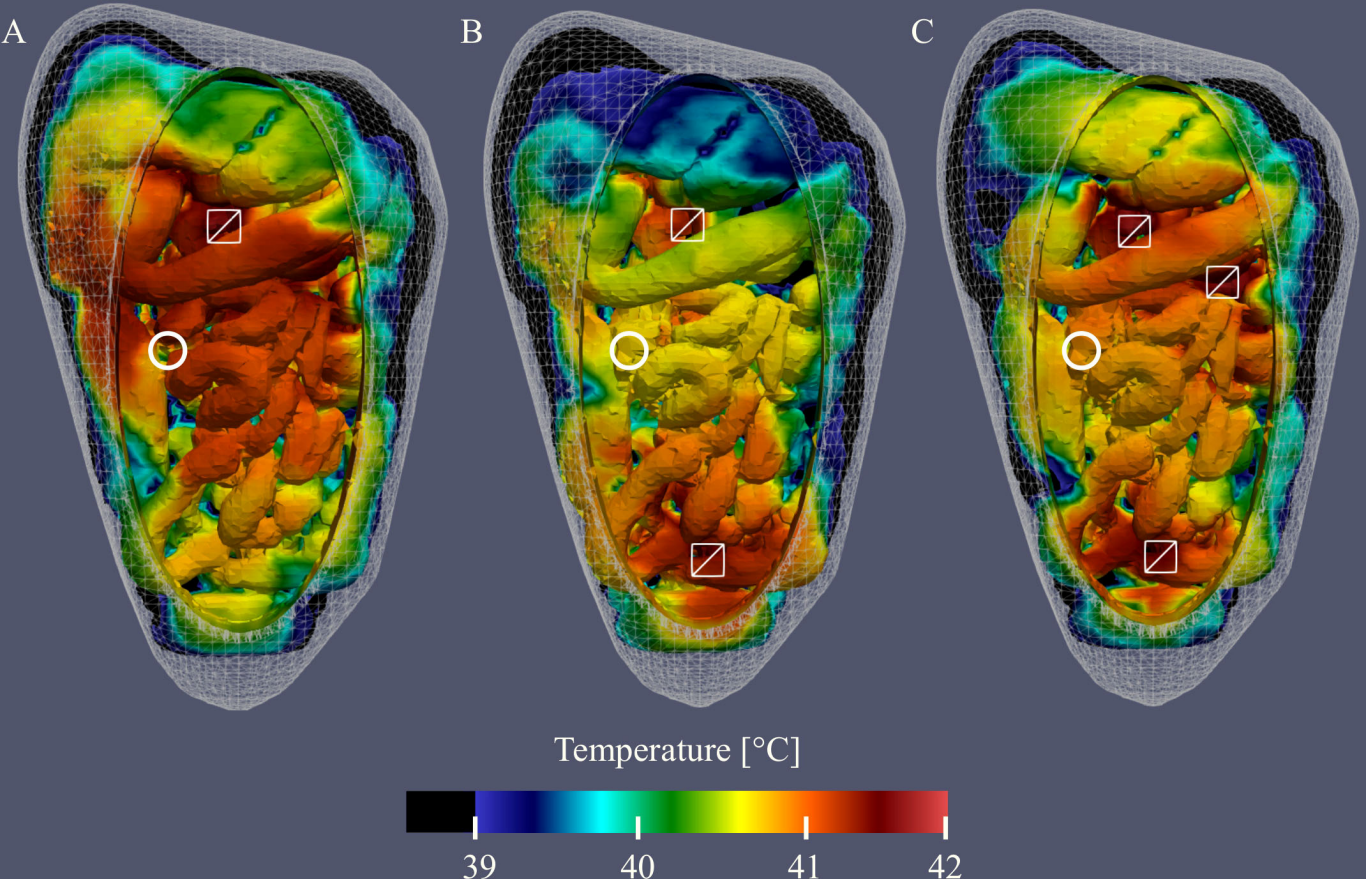
Visualization of the simulated thermal distributions and the effect of changes in catheter setup on the distribution. White squares denote the location of the inflow catheter, white circles denote the location of the outflow catheter and temperatures below 39°C are black. The thermal distribution of case #1 **(A)** shows that using one inflow at high flow rate results in high temperatures near the inflow and surroundings. Addition of 1 **(B)** or 2 **(C)** extra inflow catheters results in a more homogeneous overall distribution.

Varying catheter positions or adding catheters is an excellent tool that can be used to influence the thermal distribution during HIPEC. This is visualized in **Figures 9A–C** where we show the thermal distribution for 1, 2 and 3 inflow catheters, respectively. Note that the total flow rate was kept constant for these three cases. Therefore, the flow rate per catheter is split two-ways and three-way for cases #6 (B) and #7 (C), respectively. This has a significant effect on the reach of the flow, lowering the treatment temperature near regions distant from the inflow catheter(s). Nevertheless, **Figure 9** shows that the addition of catheters can result in a better distribution, with less heat accumulating in a certain region, making it an excellent tool for flow optimization.

In **Table 6** we compare experimental and simulated values for 1, 2 and 3 catheters setups. The first three columns show the absolute errors between simulation and experiment in the last 5 minutes, i.e. “steady state”, per region. The last 3 columns show the overall absolute errors per region. All values below 0.5°C are denoted in boldface. In the last 5 minutes, all average absolute errors per region were below 0.5°C. On average, the absolute errors were below 0.3°C. Errors were larger incorporating the entire duration of the experiment because of large(r) errors occurring in the first half of the experiment, where gradients were large(st). However, average absolute errors were still relatively low with 0.5, 0.6 and 0.65°C for the 1, 2 and 3 inflow catheters setup, respectively. Errors were larger for an increasing number of inflow catheters. This is due to the increased uncertainty that is introduced by each inflow catheter. The distance between measurement points and inflow catheters also decreases for an increased number of inflow catheters. Measurement points near inflow catheters are more dependent on inflow variations and are therefore more prone to variations during measurement; this is also visible in **Table 3** and **Figure 4B** for regions 1 and 3.

**Table 6:** Absolute errors between predicted an measured values for catheter variations (cases #1, #6 and #7).

	Steady state			Overall		
No inflow catheters	1	2	3	1	2	3
Outflow [°C]	**0.10**	**0.13**	**0.06**	**0.27**	**0.37**	**0.24**
Region 1 [°C]	**0.37**	**0.45**	**0.37**	1.11	0.77	0.89
Region 2 [°C]	**0.33**	**0.35**	**0.41**	1.16	0.65	0.95
Region 3 [°C]	**0.41**	**0.38**	**0.45**	0.65	1.25	1.02
Region 4 [°C]	**0.24**	**0.36**	**0.39**	0.59	0.65	**0.48**
Region 5 [°C]	**0.07**	**0.13**	**0.27**	**0.12**	**0.37**	**0.3**
Region 6 [°C]	**0.08**	**0.29**	**0.36**	0.53	0.60	1.12
Region 7 [°C]	**0.09**	**0.39**	**0.20**	**0.20**	0.52	0.66
Region 8 [°C]	**0.11**	**0.31**	**0.23**	**0.31**	**0.33**	0.54
Region 9 [°C]	**0.27**	**0.13**	**0.22**	**0.24**	0.53	**0.28**
Average [°C]	**0.21**	**0.29**	**0.30**	0.52	0.60	0.65

Differences below 0.5°C are denoted in boldface. Near steady state, all values were below 0.5°C. Including the entire range, differences could exceed 0.5°C, mostly due to outliers during the first 10 minutes where temporal gradients were largest and measurement errors were highest, see **Figure 4**.

## Discussion

In this study we investigated the accuracy of the thermal module of the treatment planning software developed for HIPEC treatments (16, 24). To this end, we designed a phantom representing a life-sized female peritoneal anatomy during an open HIPEC treatment. This model was 3D-printed and used in a HIPEC setup in which we were able to generate different HIPEC treatment scenarios by varying catheter setups, flow rates and inflow temperatures. The thermal distribution was measured in 9 different regions with 7-point probes, totaling 63 measurement points in the phantom. By comparing experimental data with simulations generated by the treatment planning software, we were able to determine the absolute error to be below0.5°C for steady-state situations.

Inflow temperatures varying between 37-44°C (usually 40-43°C) are applied during HIPEC treatments (6). Although the inflow and outflow temperature are relatively easy to control, it is much more difficult to robustly control temperature distributions across the entire peritoneal surface. This has been demonstrated in *Schaaf et al.* (27) and *Rettenmaier et al.* (14). Treatment temperatures in patients do tend to reach hyperthermic levels on average, but variations of up to 4 degrees can occur between regions and patients. Therefore, with an accuracy well below 0.5°C, the treatment planning software can provide accurate estimates to help achieve hyperthermic temperatures in critical regions. When hyperthermic temperatures above 40°C are reached in the pelvic region and omental bursa for a minimal duration of 40 minutes, patients are observed to have increased overall survival and progression free survival (27). In an interesting study performed by Ye et al., a relation between thermal stability and bowel function recovery was found. The authors observed that flatus, defecation passage and enteral nutrition initiation returned 2 days faster in patients treated with a stable temperature compared to patients treated with a fluctuating perfusion temperature (11). The thermal fluctuations were defined as temperature drops in the outflow temperature. Likely, these drops are associated with accumulation of heat elsewhere in the peritoneum resulting in the adverse effects, demonstrating the importance of preventing hot spots. The three parameters discussed in this study; inflow temperature, flow rate and catheter setup can be adjusted to realize a stable and sufficiently high and uniform therapeutic treatment temperature.

The treatment planning software aims to help improve the homogeneity in the peritoneal cavity during HIPEC. This is important because the cytotoxic enhancement of the chemotherapeutics strongly depends on the temperature achieved. This enhancement is determined during preclinical *in vitro* and *in vivo* research and can be quantified by the thermal enhancement ratio (TER). Results from these experiments indicate the optimal local treatment conditions while the treatment planning software should be able to determine the optimal way to deliver and achieve these optimal conditions. Since it is difficult to generate statistically significant results for thermal variations below 0.5°C during preclinical research, prediction of temperatures with an accuracy below 0.5°C is also sufficient to provide relevant indications on local thermal enhancement during treatment.

Clinically, flow rates between 0.5 and 2 liter per minute are used (6). The influence of flow rate on intraperitoneal temperatures is straightforward. An increased flow rate causes a decrease of the time of the perfusate spent in the peritoneal cavity, directly increasing the outflow temperature. With a proper flow distribution, this can also directly increase the treatment temperature in all peritoneal regions. This phenomenon was also observed in a study by *Batista et al.* (28), wheretemperature differences between inflow and outflow decreased from 3.1°C to 1.1°C for flow rates of 600 mL/min and 1000 mL/min, respectively. We observed a similar decrease from 2.4 to 1.5°C for the same flowrates. While observing the same trend, the absolute difference between (28) and our results is likely caused by the lower inflow temperature for high(er) flow rates in the former study. Another important consequence of high flow rates is the earlier onset of a steady-state. In an experimental study, *Furman et al.* observed that the time for a saline bag, functioning as viscera, to reach 43°C was 55, 40, 35 and 25 minutes for flow rates of 1, 2, 3 and 4 L/min, respectively. In our simulation and experimental model it took about 5 minutes less to achieve a steady state, defined as Δ*T*<0.1 °C, for 1000 mL/s compared to 600 mL/min. A higher flow rate can also impact the core temperature. In previous studies we showed that higher flow rates resulted in higher core temperatures in a rat (16, 24). This phenomenon is also to be expected in humans. Perfusion is usually started when a stable flow is realized. Changing flow rates during treatment might result in obstruction of the outflow catheter(s) by peritoneal tissues. Therefore, an appropriate flow rate has to be chosen before treatment and is limited by clinical feasibility. The software presented in this study can be used to estimate an appropriate flow rate before treatment. In **Figures 6**, **8** plus **Tables 4** and **5** we showed that a flow rate increase has a similar effect on local temperatures as an increase in inflow temperature. Both result in an absolute shift, without altering the distribution. Therefore, flow rate and inflow temperature should be optimized simultaneously to achieve an adequate treatment temperature without causing local and systemic thermal damage. To change the spatial distribution, other parameters could be adjusted, such as the catheter setup.

In **Figures 8A–C** we showed that the use of multiple inflow catheters can help to achieve high(er) treatment temperatures in regions that were not heated adequately with the baseline single inflow setup. These experiments were performed with the same flow rate as the baseline case. However, the flow rate per catheter was 50% and 33% of the total flow rate due to the addition of one or two inflow catheters, respectively. Increasing the flow rate for these cases would again cause an absolute increase in treatment temperature. Therefore, increasing the number of catheters should also require reevaluation of an adequate inflow temperature and flow rate. The optimal number of catheters depends on the total peritoneal volume. For rats, we determined that 4 inflow catheters is sufficient to create a homogeneous thermal distribution (16). In the present study, we mainly focused on validation and we did not aim to optimize the number of catheters; this is part of ongoing research at our department. Catheter variation is a strategy that is not yet commonly used during HIPEC treatments. For open HIPEC treatments, catheters can be easily repositioned during treatment. However, during a closed HIPEC, catheters need to be placed before the abdomen is closed and perfusion starts. Placement of additional catheters before the start of perfusion and varying inflow and outflow over these catheters can be an option to achieve an adequate temperature in all regions of the peritoneal cavity during a closed HIPEC treatment. This approach is particularly useful when a minimal therapeutic temperature, minimal HIPEC duration, heat-sensitive organs and/or high priority treatment locations can be identified. Our treatment planning software can be used to guide placement of the catheters.

An important next step will be further clinical validation of the treatment planning software. In a clinical setting additional aspects such as organ motion and blood perfusion can play a role in the formation of the thermal distribution. *In vivo* models provide a preclinical setting in which detailed thermal measurements can be taken while incorporating the effects of perfusion and organ motion such as intestine peristalsis. In a previous study, we incorporated perfusion in an *in vivo* rat model and validated temperature predictions (24). Similar experiments could be designed for either large *in vivo* models (such as pigs) or in humans. The findings of these studies can then be translated towards human applications. For clinical applications, patient specific anatomical features should be included to ensure reliable and relevant predictions. Ideally, geometries would be generated from a patients’ computed tomography (CT) or magnetic resonance (MR) images. However, given the complexity of the model, developing a well-detailed model for each individual patient separately is presently a very time consuming task and is therefore presently not feasible in a clinical workflow. Instead, categorizing patients based on body mass index or body surface area could provide accurate standardized models reflecting a patients’ spatial characteristics. Furthermore, modified models could also incorporate large organ resections that could result in additional peritoneal space. This way, the treatment planning software can be expected to help optimization of future HIPEC treatments.

The accuracy demonstrated in this study is sufficient to help optimize treatment strategies and guide HIPEC treatments on how to achieve an optimal thermal distribution. Simulations can detect how to target certain regions by adequate catheter placement or addition of catheters and achieve therapeutic peritoneal temperatures by adjusting flow rate and inflow temperature. Another interesting tool that can be used to optimize homogeneity is flow inversion which is achieved by changing inflow to outflow and vice versa. By varying patient characteristics, catheter setup, flow rate and inflow temperature, a library of plans can be generated to guide HIPEC treatments. In combination with adequate temperature monitoring in key locations, live feedback can be provided suggesting if and which alterations are needed during treatment. The study presented is an important step towards the clinical application of the software. Our next step will be the clinical translation of both the software and the phantom by validating the thermal distribution on clinical data. When successful, the software can be employed for evaluating several treatment strategies with the goal of optimizing HIPEC treatments.

## Summary and conclusions

In this study, treatment planning software for HIPEC was validated using a realistic life-sized 3D printed phantom representing the female peritoneum. We used this phantom in an experimental HIPEC setup in which we varied the flow rate, inflow temperature and number of catheters. The temperature was measured at 63 locations in the phantom. Increased flow rates and inflow temperature resulted in an absolute shift in local treatment temperature while increasing the number of catheters improved the overall homogeneity. The simulations performed with the treatment planning software compared well with the experiments and predicted the temperature shifts. For all cases, absolute errors remained below 0.5°C for steady-state situations. Temporal behavior was more difficult to predict, with errors around 0.5°C. Based on clinical data, showing possible temperature variations of up to 4°C, these predictions are sufficiently accurate to provide important estimates on local temperature variations and to optimize HIPEC treatment setups to improve clinical outcomes.

## Data availability statement

The raw data supporting the conclusions of this article will be made available by the authors upon request.

## Author contributions

Conceptualization, HK, JC, and DL; methodology, DL; formal analysis, DL; investigation, DL; data curation, DL; writing—original draft preparation, DL; writing—review and editing, DL, RH, HK, JC, NF, AO, PT, JT, RZ, and JS; visualization, DL; supervision, JC, HK, and PT; project administration, JC; funding acquisition, JC, NF, HK, and PT. All authors contributed to the article and approved the submitted version.
